# Attrition in the kimberlite system

**DOI:** 10.1007/s00710-018-0580-0

**Published:** 2018-05-18

**Authors:** Thomas J. Jones, James K. Russell

**Affiliations:** 1 Department of Earth Sciences, Durham University, South Road, Durham, DH1 3LE UK; 2 Department of Geosciences, University of Tuebingen, Wilhelmstrasse 56, 72074 Tuebingen, Germany; 3 Department of Earth, Ocean & Atmospheric Sciences, University of British Columbia, Vancouver, BC V6T 1Z4 Canada

**Keywords:** Kimberlite ascent, Olivine fines production model, Olivine wear, Abrasion, Milling, Crystal breakage

## Abstract

The sustained transportation of particles in a suspension commonly results in *particle attrition* leading to grain size reduction and shape modification. Particle attrition is a well-studied phenomenon that has mainly focussed on sediments produced in aeolian or fluvial environments. Here, we present analogue experiments designed to explore processes of attrition in the kimberlite system; we focus on olivine as it is the most abundant constituent of kimberlite. The attrition experiments on olivine use separate experimental set-ups to approximate two natural environments relevant to kimberlites. Tumbling mill experiments feature a low energy system supporting near continual particle-particle contact and are relevant to re-sedimentation and dispersal processes. Experiments performed in a fluidized particle bed constitute a substantially higher energy environment pertinent to kimberlite ascent and eruption. The run-products of each experiment are analysed for grain size reduction and shape modification and these data are used to elucidate the rates and extents of olivine attrition as a function of time and energy. Lastly, we model the two experimental datasets with an empirical rate equation that describes the production of daughter products (fines) with time. Both datasets approach a fines production limit, or plateau, at long particle residence times; the fluidized system is much more efficient producing a substantially higher fines content and reaches the plateau faster. Our experimental results and models provide a way to forensically examine a wide range of processes relevant to kimberlite on the basis of olivine size and shape properties.

## Introduction

Attrition, driven by particle-particle interactions, results in the size reduction and shape modification of particles and operates in a wide variety of geological environments. From the Engineering Sciences, we identify two modes of attrition that can operate within a gas/liquid suspension (Bemrose and Bridgwater 1987; Xiao et al. 2014; Jones et al. 2017): (1) fragmentation and (2) abrasion. Fragmentation involves complete fracturing of the original particles (i.e. parent particles) to form a group of smaller, daughter particles. ‘C*ritical*’ collisions causing fragmentation typically occur through direct high-energy impact with other particles or with a hard surface at, or above, a threshold velocity (e.g. Dufek et al. 2012). Abrasion is a less energetic process wherein the rough edges or asperities of the parent (and daughter) particles are rounded off to produce particles with smoother surfaces and morphologies. A consequence of abrasion processes is parent grain size reduction by the production of very fine particles, often measuring ≤10 μm.

There are several factors that control the rates of attrition and they can be broadly divided into two groups: particle properties and environmental factors (Bemrose and Bridgwater 1987). Particle properties include, but are not limited to size, shape, surface texture, size distribution, hardness, and the presence (or absence) of internal defects. Environmental factors affecting rates of attrition include particle residence times, particle concentrations, temperature, pressure, and differential velocities between colliding particles or between particles and a confining wall. Attrition is most successful when differential velocities are high, particle concentrations are high, parent particle shapes are irregular, residence times are long and particle size distributions are poorly sorted (e.g. Forsythe and Hertwig 1949; Bemrose and Bridgwater 1987; Ray et al. 1987; Xiao et al. 2011; Knight et al. 2014; Xiao et al. 2014; Jones et al. 2017).

**Fig. 1:**
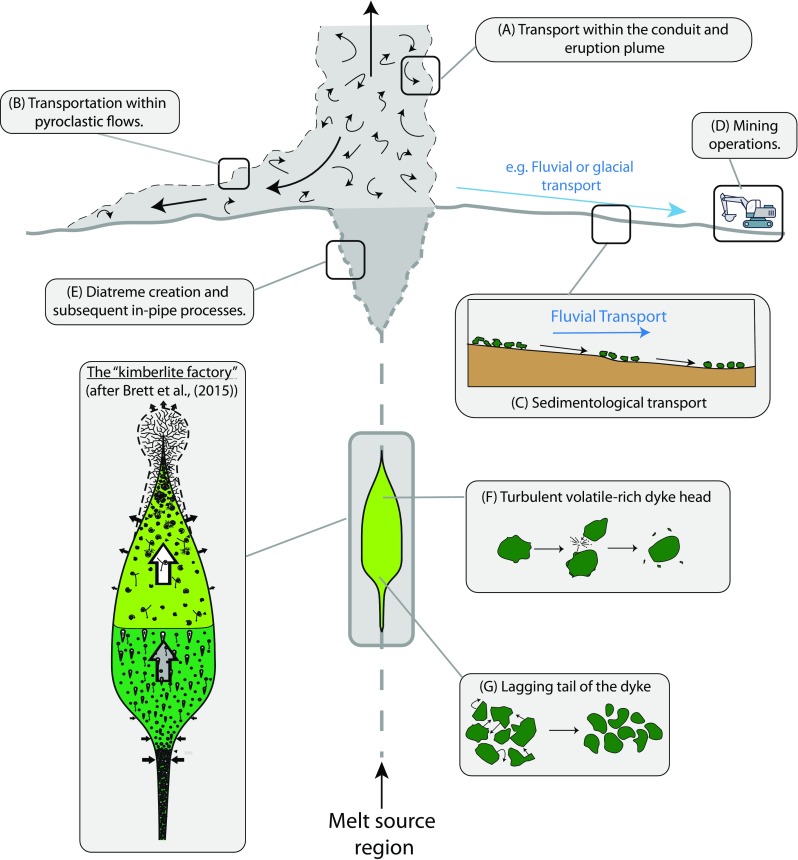
Schematic diagram of the “kimberlite system”, illustrating where attrition processes can operate. **a** Transport within the conduit and eruption plume supports particle attrition. **b** Transportation within pyroclastic flows. **c** Low energy particle-particle interactions in the form of sedimentological bedload transport (e.g. traction). **d** Mining operations. **e** Creation of the diatreme and subsequent eruption and remobilisation events within the pipe. **f** Turbulent volatile-rich dyke head wherein xenocrysts are subject to a high energy, high velocity, turbulent environment. **g** Lagging tail of the dyke wherein further grain rounding may occur at relatively low energy, with particles in near-continual contact

Particle attrition affecting kimberlitic minerals operates in several different environments between the lithospheric mantle and the site of kimberlite extraction on the Earth’s surface (Fig. 1). Mantle-derived olivine is the dominant constituent of kimberlite (Mitchell 1986; Mitchell 1995) and commonly shows strong evidence for reshaping during transport, eruption and re-sedimentation (Reid et al. 1975; McCandless 1990; Arndt et al. 2006; Afanas’ev et al. 2008; Holden et al. 2009; Jerram et al. 2009; Arndt et al. 2010; Moss et al. 2010; Moss and Russell 2011; Jones et al. 2014; Brett et al. 2015). On that basis, our study focusses on olivine attrition. Olivine has poor cleavage and often displays brittle conchoidal fracture. Attrition of olivine occurs in many parts of the “*kimberlite system*” (Fig. 1) and has import for (1) tracing of indicator minerals in reworked and transported kimberlite materials, (2) interpretation of volcaniclastic deposits of kimberlite based on grain size and shape analysis, (3) understanding diamond grade distributions in volcaniclastic kimberlite deposits (Harvey et al. 2013), and (4) establishing the conditions (transport regime and duration) attending kimberlite transport during ascent through the lithosphere.

The ascent of kimberlite magma is considered to be fast relative to most other terrestrial magmas with estimates varying between ~1 to 10’s m s^−1^, corresponding to total average transit times of <10 h to ~2 days (McGetchin et al. 1973; Eggler 1989; Sparks et al. 2006; Wilson and Head 2007). There is strong evidence and general acceptance that the buoyancy supporting rapid ascent derives from the presence of an exsolved fluid (e.g. Russell et al. 2012; Sparks 2013; Stone and Luth 2016). The exsolution of a low-density volatile phase reduces the magma’s bulk density, provides buoyancy, and promotes accelerated ascent creating fast-moving, potentially-turbulent, suspensions of melt, fluid and mantle cargo (e.g. Sparks et al. 2006; Wilson and Head 2007; Arndt et al. 2010; Russell et al. 2012). As advanced by Jones et al. (2014) and Brett et al. (2015), this environment, attending the transport of kimberlite magma, is ideal for efficient attrition of the abundant mineral grains liberated by mantle xenolith breakup. They suggested that the rounded shapes and abraded surfaces of mantle-derived xenocrystic minerals are a strong indicator of turbulent ascent of kimberlite magmas through the lithospheric mantle and within subsurface feeder dykes. However, the exact depths at which CO_2_ saturation occurs in the mantle lithosphere remains a matter of debate and is highly dependent on many intrinsic variables such as the melt composition, temperature and original CO_2_ content (e.g. Eggler 1989; Wilson and Head 2007; Russell et al. 2012; Brett et al. 2015; Stone and Luth 2016; Stamm and Schmidt 2017).

Rapid turbulent transport of kimberlite through the mantle lithosphere represents an important environment for physical modification of xenocryst shapes, sizes and surfaces. However, xenocryst properties will continue to be modified during emplacement and eruption and during and after kimberlite deposition. Pipe forming and pipe filling phases of eruption produce a wide range of volcaniclastic lithofacies including primary pyroclastic vent-proximal deposits to significantly reworked kimberlite-rich sediments (e.g. Stiefenhofer and Farrow 2004; Brown et al. 2009; Porritt and Cas 2009; Buse et al. 2011; Porritt and Cas 2011; Sparks 2013).

The explosive phase of kimberlite eruption can result in olivine fragmentation and, in fact, the shapes of olivine (e.g. fractured) can be critical elements to identifying pyroclastic deposits (Moss et al. 2008; Buse et al. 2011; van Straaten et al. 2011). High energy explosive eruption environments occur during: i) the onset of the eruption (e.g., brecciation of near-surface crustal rock), ii) the pipe excavation phase of eruption (e.g. Brown et al. 2008; Barnett 2008), iii) rapid decompression and continued volatile expansion in the conduit (e.g., sustained eruption), iv) the generation of dilute surge currents (e.g., phreatomagmatic explosive events; Porritt et al. 2013), and v) during the fluidization of pyroclastic infill (e.g., massive volcaniclastic kimberlite) (Sparks et al. 2006; Walters et al. 2006; Gernon et al. 2009b). Volatile expansion in the conduit creates a rapidly rising, turbulent, fluid-particle suspension that supports entrainment of juvenile pyroclasts, mantle crystal cargo and accessory lithic fragments into the eruption plume. This represents another, environment for particle attrition (Dufek et al. 2012; Jones and Russell 2017). More importantly, collapse of the eruption plume can generate pyroclastic density currents and/or surges (Moss et al. 2008; Porritt et al. 2008; Gernon et al. 2009a; Porritt and Cas 2011; Sparks 2013) which are high-energy environments capable of particle reshaping and resizing. Previous studies of attrition processes in silicic pyroclastic density currents (PDCs) have documented the effects of attrition on pumice during transport (Manga et al. 2011; Kueppers et al. 2012), and the abrasive stripping of glass from crystal surfaces (Meyer 1971; Jones et al. 2016). Logically, these studies have shown attrition rate to decrease with distance from the vent, reflecting the dissipation of energy with transport distance (Dufek and Manga 2008).

Kimberlite deposits and the components within the deposits are all susceptible to post-eruption processes (e.g. re-sedimentation). Attrition of kimberlite indicator minerals during transport within glacial and fluvial systems is of interest to diamond exploration because of its capacity to inform on distances to the source kimberlite pipe (McCandless 1990; Afanas’ev et al. 2008; Afanasiev et al. 2011; Afanasiev and Pokhilenko 2013). Several experimental studies have established the relative attrition susceptibility of different kimberlite indicator minerals (e.g., garnet, ilmenite, pyroxene) by using experimental duration as a proxy for transport distance. Using an ultrasonic dispenser Afanas’ev et al. (2008) were able to quantify the relative abrasion stability of different mineral phases. In decreasing stability, they report the following order: diamond, pyrope, olivine, picroilmenite, apatite, kimberlite (rock fragments). Cummings et al. (2014), experimentally abraded pyrope, chrome diopside and ilmenite grains in a tumbling mill and found that the pyrope grains abraded the easiest and in two stages. Firstly, by the removal of a kelyphite rim by surface abrasion and then, secondly, by grain breakage (fragmentation) along internal defects. Furthermore, the concept of grain size modification and the inherent particle size distribution evolution accompanying fluvial transport has been used to evaluate the diamond grade in placer-style deposits (e.g. Sutherland 1982). Lastly, we note that, grain size reduction by attrition can occur during mining and extraction processes and often contributes a significant economic loss via the breakage of diamonds (Armstrong 2017).

Within any geological environment, the nature of particle-particle interactions can be highly variable. However, to a first-order, attrition operates as two contrasting styles: (1) lower energy sustained abrasive contacts and (2) high velocity, transient particle-particle or particle-wall interactions. Here, we use experiments on olivine in a tumbling mill and a fluidized bed, respectively, to investigate and compare these two styles of attrition. Our experiments focus on the attrition of olivine, the dominant mantle phase in kimberlite, however, the fundamental principles we address here are pertinent to the attrition of all mantle-derived phases.

## Methods

### Pre-experiment sample characterization

Commercially-produced olivine sands, extracted from the mining of dunite, were purchased from the United Western Supply Company, Seattle (Uniwest), and from Luossavaara-Kiirunavaara Aktiebolag (LKAB) minerals (see Tables 1 and 2). Prior to experimentation, the olivine sand was washed to remove any adhering fine fragments left over from production and dry-sieved to two different restricted ranges of grain size: 1–2 mm (Uniwest olivine) and 250–500 μm (LKAB olivine). After this initial preparation, an aliquot of olivine grains from these size fractions was measured for density. We used a Micrometrics Accupyc II 1340 Helium Pycnometer to measure the sample volume and an analytical balance to measure the mass. Using this technique, we report olivine grain density values of 3256.9 kg m^−3^ and 3304.9 kg m^−3^ for the Uniwest and LKAB olivine, respectively. We also used the chemical compositions determined by X-Ray florescence (ALS Chemex; Table 1) and mass balance calculations to estimate the optimal olivine content (wt%) and olivine composition (Fo; mol%) of the washed olivine sands (Table 2).

**Table 1:** Chemical compositions (wt%) of olivine sand used in this study

Provider	Uniwest	LKAB
SiO_2_	41.77	41.68
Al_2_O_3_	0.26	0.27
Cr_2_O_3_	0.45	0.22
FeO	9.35	8.30
MnO	0.12	0.1
MgO	47.9	49.7
CaO	0.19	0.05
Na_2_O	0.12	0.06
K_2_O	0.01	0.02
Total	100.17	100.40

Bulk XRF analysis by ALS Chemex

**Table 2:** Model olivine wt% and composition (Fo) in commercial “olivine sand” used in this study and residual sum of squares (RSS) on model

Provider	Uniwest	LKAB
Fo [mol%]	89.8	91.2
Olivine [wt%]	99.2	99.8
RSS	2.69	0.78

### Attrition experiments

**Fig. 2:**
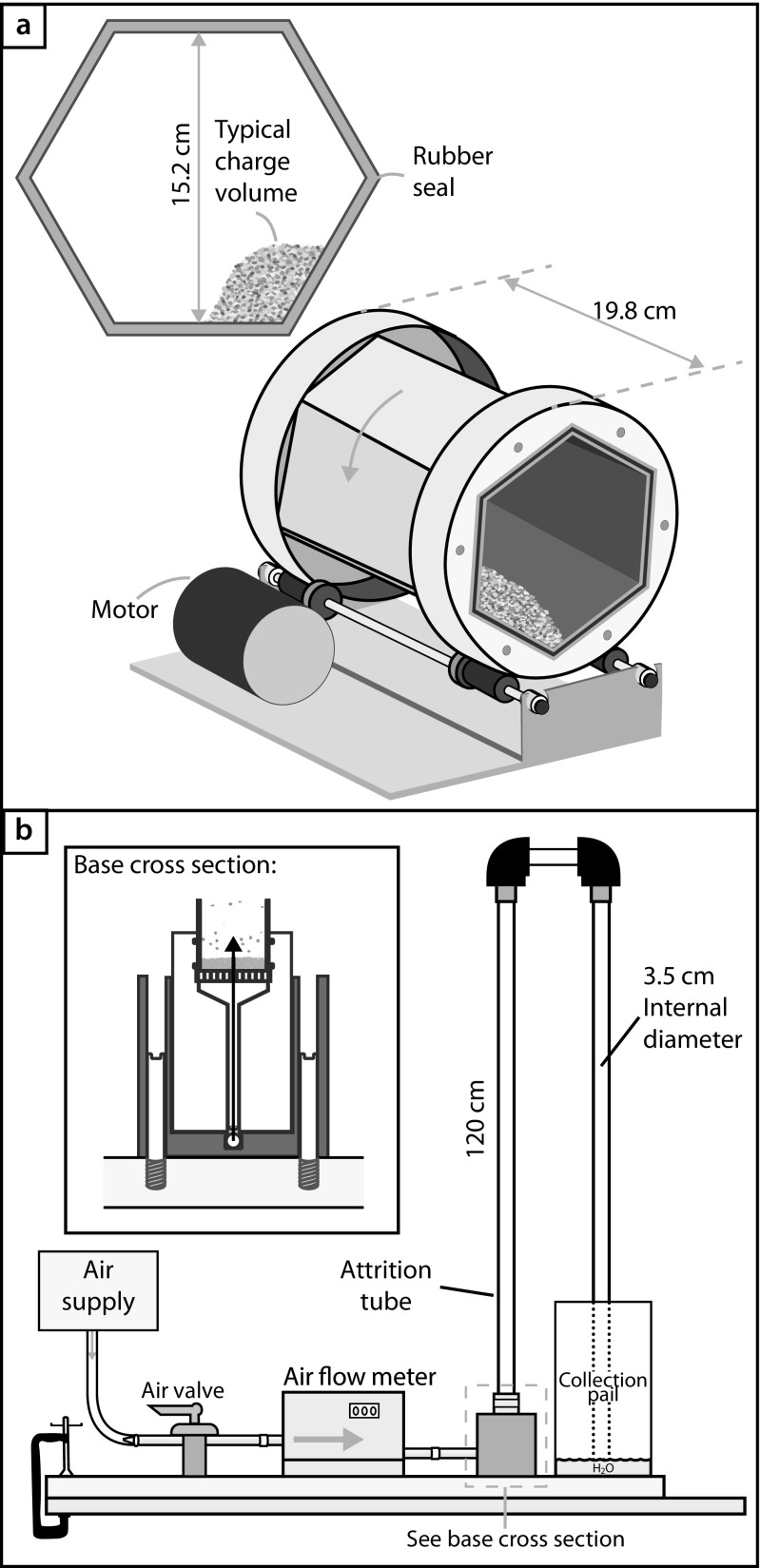
The experimental apparatuses used in this study: (**a**) Tumbling mill: a rotating mill with a hexagonal cross-section and closed on one end by a transparent window and a rubber seal. The drum measures 15.2 cm in diameter and was loaded (charged) with 120 g of Uniwest olivine in this study. The approximate loading volume is drawn in the inset. **b** Fluidized bed: gas is supplied from a compressed air supply through a calibrated flow meter, at a user-determined flow rate (85.5 L min^−1^ in this study), into the “attrition tube” where the particle-particle interactions take place. A series of tubes, connectors and a water reservoir ensure no run-product is lost and all the sample can be recovered

In this study, two types of attrition experiments were performed providing two distinct types of particle-particle interaction. Firstly, experiments were performed in a tumbling mill (Fig. 2a), where all of the input particles (feed) remain in near continual contact. In each of these experiments 120 g of Uniwest olivine was loaded into the drum of the tumbling mill and sealed to ensure no particles could escape. The drum was then placed on the roller bars and left for a prescribed amount of time rotating at 32 revolutions per minute (rpm). We conducted experiments in the tumbling mill for durations of 0.25, 0.5, 1, 2 4, 5.28, 7.22, 10, 21 and 29.83 days.

Secondly, experiments were performed in a fluidized bed (Fig. 2b), wherein the particles were turbulently suspended in a gas jet. This apparatus consists of a feeding compressed air gas supply passed through a calibrated FMA5526 Omega gas flow meter into the base of the vertical attrition tube. At the tube base (inset; Fig. 2b), there is a distributor plate containing ~ 40 evenly spaced holes to break-up the gas and provide an even flux suspending the particles above. The particle-particle interactions occur in the 120 cm long vertical transparent plastic attrition tube situated above the distributor plate; the vertical attrition tube is connected to an elbow joint with a downward return to a water reservoir designed to ensure that no fines escape the system. In this study, 70 g of (LKAB) olivine grains were loaded onto the distributor plate and, then, subjected to a gas feed of 85.5 L min^−1^ for times of 0.5, 1, 2, 4.5, 6.3, 12.5, 24 and 32 h.

### Post-experiment sample characterization

At the end of each experiment all the experimental run products were carefully collected and sieved using a standard stack of Tyler sieves. After each experiment, the apparatuses were flushed with deionised water over a 63 μm sieve to ensure that all of the fine particles were captured. The grain size distributions of all particles <63 μm, suspended in the wash water, were measured using a Malvern Mastersizer 2000 with the Hydro 2000Mu water dispersion module attached. In order to convert the volumetric measurement made by the Mastersizer to mass values (g), a known amount of 63–125 μm olivine was added to the sample. Using a pump speed of 1900 rpm, a refractive index of 1.7 and an absorption coefficient of 0.1, an aliquot of the attrition sample was added to the dispersion module and measured three times. An ultrasonic pulse was applied to the sample for 2 s before the measurement to prevent particles from aggregating in the water suspension. For each experimental product, three separate aliquots were taken, each measured three times.

To document the morphology of the olivine grains and how they change during attrition we analyzed the largest grain size fraction from both the tumbling mill (1–2 mm) and the fluidized bed (250–500 μm) experiments. For each controlled experiment (i.e. time and condition; Table 3) between 10 and 20 grains were selected from each run product and mounted for study of grain shapes and surfaces. Using a Hitachi SU-70 scanning electron microscope (SEM) and a 5 kV accelerating voltage, scanning electron images were taken at a range of magnifications.

**Table 3:** Summary of experiments conducted in this study

Type of experiment	Fo [mol%]	Input size range [mm]	Input mass [g]	Duration [h]
Tumbling mill	89.8	1—2	120.01	6.0
	89.8	1—2	120.02	12.0
	89.8	1—2	120.03	24.0
	89.8	1—2	120.06	48.0
	89.8	1—2	120.01	96.0
	89.8	1—2	120.34	126.7
	89.8	1—2	120.32	173.3
	89.8	1—2	120.02	240.0
	89.8	1—2	120.01	504.0
	89.8	1—2	118.58	715.9
Fluidized bed	91.2	0.25—0.5	69.9	0.5
	91.2	0.25—0.5	69.9	1.0
	91.2	0.25—0.5	70.1	2.0
	91.2	0.25—0.5	70.0	4.5
	91.2	0.25—0.5	70.5	6.3
	91.2	0.25—0.5	70.4	12.5
	91.2	0.25—0.5	69.9	24.0
	91.2	0.25—0.5	70.1	32.0

For the tumbling mill experiments we used images of grains >1 mm to track the change in grain shape as a function of residence time. Specifically, 150 grains from the feeding size class (1–2 mm) were randomly selected and individually photographed under a binocular microscope with an intense backlight. Then, using the software, ImageJ (http://rsbweb.nih.gov/ij/) a threshold was applied to the images to separate the grain from the image background. The images were then converted to binary masks. This 2-D projection of the grain was quantified for circularity which serves as a metric for extent of abrasion:

1 $$ \mathrm{Circularity}=4\uppi \mathrm{A}/{\mathrm{P}}^2 $$

where A [mm^2^] is the projected area and P [mm] is the grain perimeter. This shape factor is bounded such that values of 1 represent a perfect circle.

## Results

### Tumbling mill experiments

**Fig. 3:**
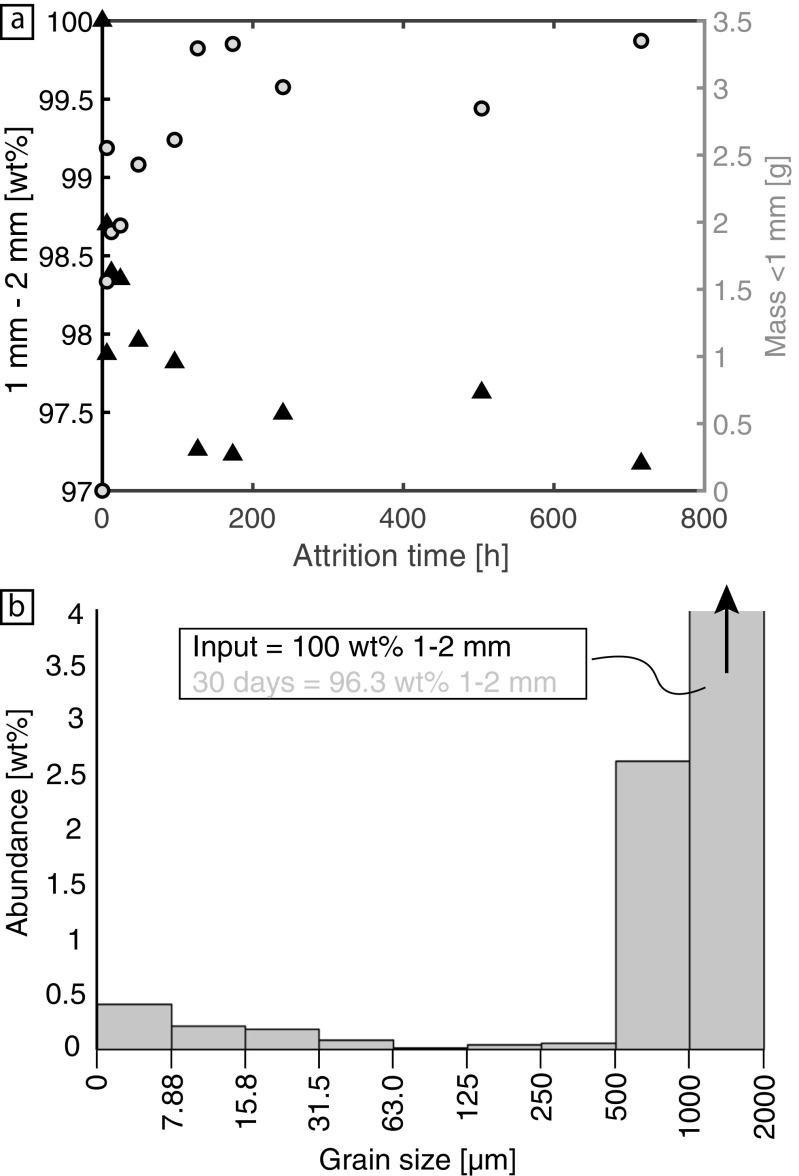
**a** Results of the grain size measurements on products from the tumbling mill. Both the percentage mass remaining in the feed grain size bin (1–2 mm; black triangles; left axis) and the mass of daughter products, i.e. grain sizes smaller than the feed (grey circles; right axis), are plotted against the attrition time in hours. **b** Grain size distribution of the run products from the 30 day experiment (longest duration investigated), with reference to the input feed

Grain size distribution changes within the tumbling mill run-products are shown in Fig. 3. The parent feed abundance (1 to 2 mm) is systematically reduced with increased residence time in the tumbling mill (black triangles; Fig. 3a). However, this reduction is non-linear with respect to time; the grains experience a rapid period of attrition within the first ~5 days and thereafter attrition appears to slow once the input grains have been reduced by ~2.5 wt%. The mass of daughter products (fragments <1 mm in diameter) increases systematically, also in a non-linear manner, with increased attrition time (grey circles; Fig. 3a). After long residence times (>5 h) the mass of daughter products does not increase and reaches a stable value of ~ 3.2 g. In some cases, the parent mass decay and subsequent increase in daughter abundance is not systematic (e.g. data at 504 h; 21 days), this may result from small olivine fragments adhering to these larger grains. By comparing the restricted 1–2 mm grain size range of the input (parent) material to the experimental run products after 30 days (the longest duration investigated), it can be seen that grains 500 μm to 1 mm in diameter contribute most of the daughter product mass (Fig. 3b). Furthermore, a secondary mode is observed at grain diameters <7.88 μm.

**Fig. 4:**
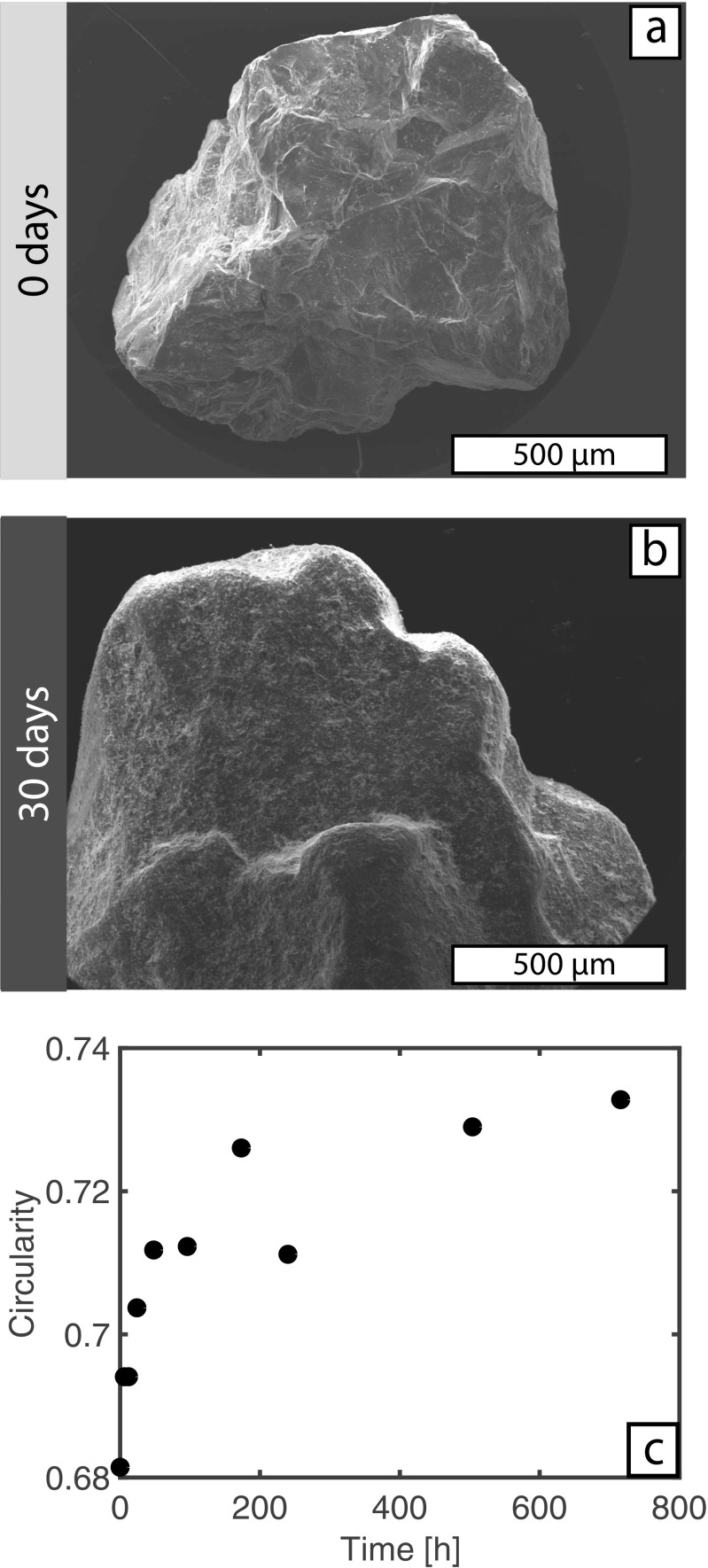
Shapes and surfaces of the olivine grains attritted in the tumbling mill. **a** Scanning electron image of a starting feeding (0 days) olivine grain. **b** Scanning electron image of an olivine grain after 30 days of attrition in the tumbling mill. **c** Quantification of how olivine grain circularity changes with increased attrition

The original olivine grains have an angular morphology and most external surfaces show conchoidal fracture (Fig. 4a). These initial features are inherited from the mining process wherein the dunite rocks are crushed and sieved to a range of industrial size classes. The starting material properties are similar to, although not identical, to the mantle material liberated at the crack-tip of the ascending dyke (e.g. Jones et al. 2014; Brett et al. 2015). In the tumbling mill experiments these sharp exterior edges become increasingly rounded with time and small olivine chips/fragments are observed to adhere to the grain surface. The surfaces of the grains become rough at the micron scale (~10’s of μm) and feature multiple hemi-spherical chips and pits (Fig. 4b). We used image analysis to quantify the changes in morphology as a function of attrition time (Fig. 4c). The original grains have a starting circularity value of 0.681 and, as they become rounded, their circularity increases with residence time. Similar to the grain size data (Fig. 3), the shape modification is exponential with respect to time. In the first ~7 days grains rapidly round to reach a circularity of 0.726, after which the rate of rounding decreases and after a further 23 days of tumbling circularity values are only marginally increased (0.733). We note that the anomalous data point at 240 h (10 days) can be correlated with a small (0.2 g) loss of material during the experiment.

### Fluidized bed experiments

**Fig. 5:**
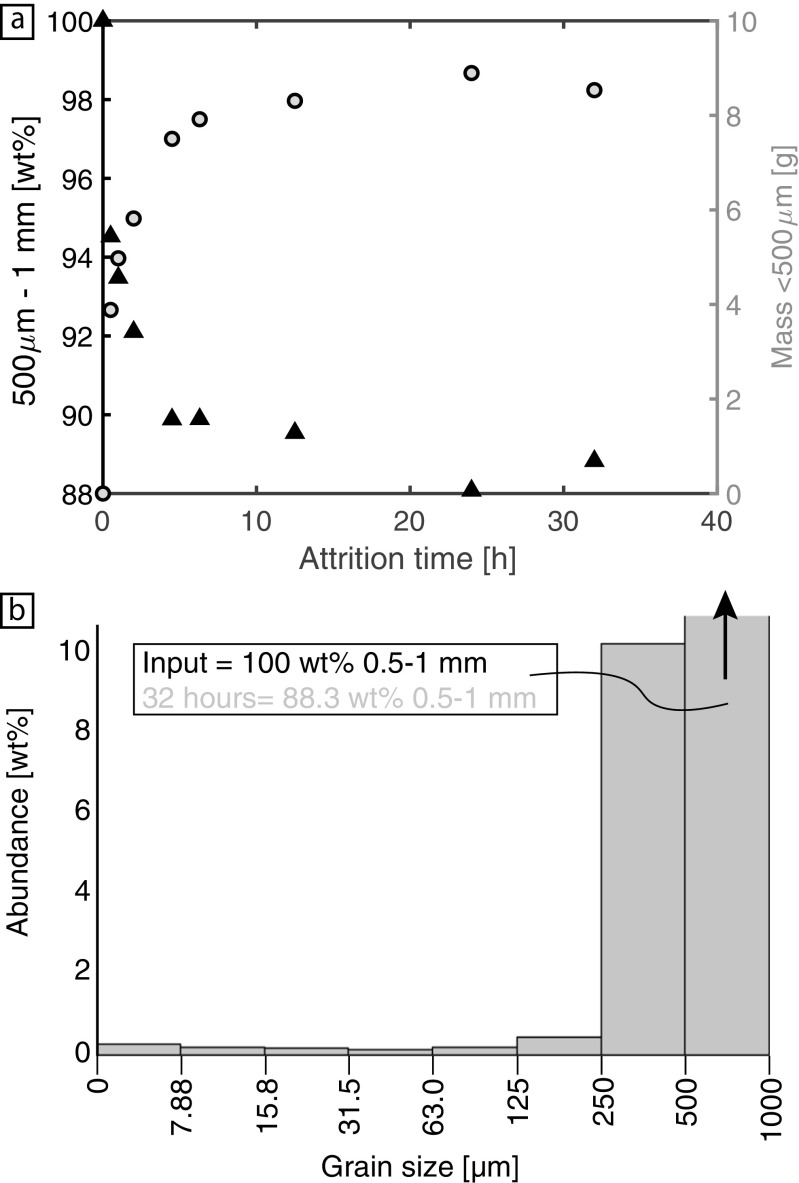
**a** Results of the grain size measurements on products from the fluidized bed. Both the percentage mass remaining in the feed grain size bin (500 μm - 1 mm; black triangles; left axis) and the mass of daughter products, i.e. grain sizes smaller than the feed (grey circles; right axis), are plotted against the attrition time in hours. **b** Grain size distribution of the run products from the 32 h experiment (longest duration investigated), with reference to the input feed

The grain size data from the fluidized bed experiments are presented in the same way as the tumbling mill datasets (Fig. 5). Again, the parent feed abundance (initially 70 g of 500 μm to 1 mm) is systematically reduced in an exponential manner with increased residence time (black triangles; Fig. 5a). The period of rapid, initial attrition occurs within the first 4.5 h and reduces the feed abundance to ~90%. Also, the mass of daughter fragments produced (grey circles; Fig. 5a) rapidly increases to 7.4 g within the first 4.5 h and thereafter reaches a stable value of ~8.0 g. Again, to observe how the grain size distribution changes with attrition, the grain size distribution of the run products from the longest duration experiment (32 h) is plotted with reference to the initial feed (Fig. 5b). Grains with diameters between 500 and 250 μm constitute most of the daughter mass (~10 wt%) and like with the tumbling mill data a second mode is observed at <7.88 μm.

**Fig. 6:**
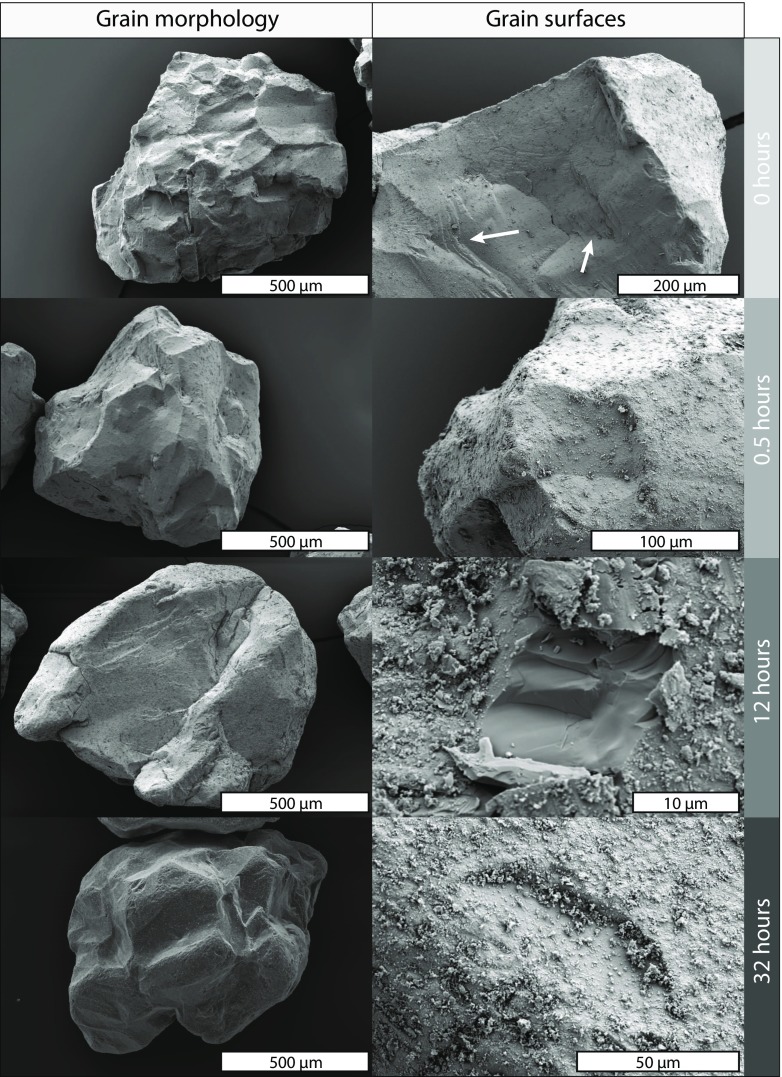
Scanning electron images of the shapes (left) and surfaces (right) of the olivine grains attritted in the fluidized bed. Residence times are denoted by the labels on the right

Morphological changes were documented via scanning electron microscopy for olivine grains measuring between 500 μm to 1 mm in diameter. The overall grain morphology (left panel; Fig. 6) progressively changes from an irregular shape with sharp edges to a sub-rounded grain with increased attrition time. The higher magnification images document the evolution of the olivine crystal surfaces (right panel; Fig. 6). In chronological order, the parent crystals have sharp, straight exterior edges and at higher magnification, their surfaces commonly feature river lines, hackle marks and evidence of conchoidal fracture (highlighted by white arrows). After 30 min, the exterior edges of the grain become more rounded and the surfaces begin to exhibit small adhering olivine fragments. After 12.5 h, the number of adhering fragments is dramatically increased and signs of surface fracture become abundant. An example of this is the central pit found in the 12.5 h olivine (Fig. 6). This fracture is interpreted to be recent as there are no adhering fine particles. Lastly, after 32 h the pits and chips also become smoothed and coated in small olivine fragments. These fine particles that commonly adhere to larger grains are thought to account for any non-systematic variations in grain size (Fig. 5).

## Analysis and discussion

### Rates of attrition

Both our datasets show that attrition is non-linear with respect to time. This is a well-known phenomenon and was first modelled by Gwyn (1969) as

2 $$ {\mathrm{m}}_{\mathrm{d}}/{\mathrm{m}}_0={\mathrm{kt}}^{\mathrm{x}} $$

where m_d_ is the mass of daughter particles, m_0_ the mass of parent feed, t is particle residence time and k and x are constants. Since this early model, workers have used comminution theory to shape three energy based models named after the proponents: the Rittinger (von Rittinger 1867), Kick (Kick 1885), and the Bond (Bond 1952) models. These three models have been used with variable success to describe attrition phenomena but generally underestimate the energy required for attrition (Rhodes 2008). Furthermore, the Gwyn (1969) model produces unrealistic infinite time limits. On that basis, we use the empirical model of Jones et al. (2017)

3 $$ {\mathrm{m}}_{\mathrm{d}}/{\mathrm{m}}_0=\mathrm{a}\ \left(1\hbox{--} {\mathrm{e}}^{\hbox{--} \mathrm{bt}}\right) $$

where a is the infinite-time attrition limit and b is the attrition rate constant [h^−1^]. Eq. 3 predicts attrition as a function of time for a model where the energy input into the system remains constant (e.g. the rotation rate of the tumbling mill remains constant).

**Fig. 7:**
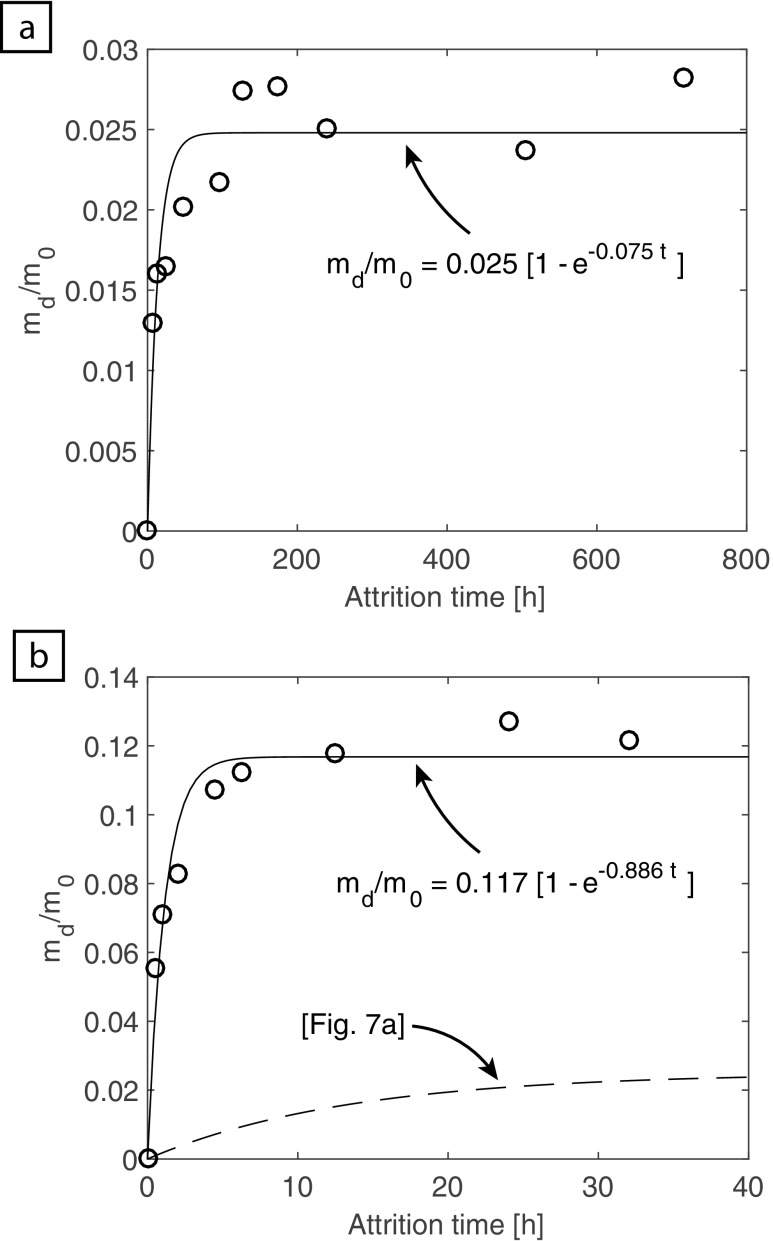
Attrition rate plots, after Jones et al. (2017), for the tumbling mill (**a**) and the fluidized bed (**b**). Where the mass ratio of daughter fragments (m_d_) to parent feed (m_0_) is plotted against the attrition time in hours. To aid comparison between the datasets, in plot (b) the tumbling mill model curve is shown by a dashed line

The results of fitting the Jones et al. (2017) model to our new experimental data are shown in Fig. 7. These models show that the tumbling mill (Fig. 7a) is capable of producing a daughter to parent mass ratio of 0.025 at infinite times and has an attrition rate constant (b) of 0.075 h^−1^. Whereas the fluidized bed (Fig. 7b) has a and b constants that are one order of magnitude larger: 0.117 and 0.886 h^−1^ respectively. We interpret this difference to result from the different conditions that the olivine grains experience. It is known that the differential velocity between particles or objects (e.g. container walls) at impact is one of the main factors governing attrition (Bemrose and Bridgwater 1987). Higher differential velocities make attrition more successful. As a comparison, we can calculate the differential velocity an olivine grain may experience in each experimental set-up. For simplicity, in both cases we assume the differential velocity to be upon contact with a static part of the apparatus. In the case of the tumbling mill, the rotation speed was measured to be 32 rpm, this corresponds to an olivine grain velocity of 0.28 m s^−1^. For the fluidized bed, videos of an experiment were taken and subsequently used to track the evolution of the particle front during an experiment. Ten repeat measurements gave an average velocity of 0.86 m s^−1^. We hypothesize that, in this case, this variation in particle velocity explains the increased attrition efficiency within the fluidized bed. However, it is important to note that differential particle velocity is not the sole factor that effects attrition. In other situations, or systems, attrition rate can be controlled by other variables. Examples include: the feeding grain size distribution; temperature and the particle concentration.

### Mechanisms of attrition

As previously stated, attrition takes two forms: fragmentation and abrasion. The grain size data from both experimental methods show that most of the size reduction occurs by rounding of the parental feed and the simultaneous of production of fines. We therefore interpret that abrasion is the main form of attrition in both the tumbling mill and the fluidized bed. This interpretation is further supported by the SEM imagery; the surfaces of the parent feed are found to have impact chips and pits that increase in abundance with increased time. Also, with increased residence time in the apparatus grains exhibit a greater number of adhering fine fragments. We interpret these fragments to be the products of abrasion. We suggest that under our experimental conditions most of the grain differential velocities are not above the “critical” velocity for fragmentation to occur (e.g. Dufek et al. 2012). Furthermore, if fragmentation dominated we would observe a sub-population of grains at an intermediate grain size – this is not observed.

In the few instances where olivine surfaces are fresh enough to be observed in the rock record they also show evidence for abrasion to be the dominant attrition process (Jones et al. 2014; Brett et al. 2015). Specifically, Jones et al. (2014) present a series of SEM images from olivine grains originally located in within a lava flow. All the grains are highly rounded and their surfaces exhibit several hemi-spherical chips and numerous pits (as observed in Figs. 4 and 6). This further supports the hypothesis that mechanical particle-particle interactions modify the mantle crystal cargo during portions of the subsurface kimberlite ascent prior to effusive lava flow emplacement. However, the experimental products from both the tumbling mill (circularity of ~0.73) and the fluidized bed do not appear as rounded as those reported from the Igwisi Hills volcano (circularity of ~0.85; Jones et al. 2014). We therefore suggest that conditions attending parts of kimberlite ascent are likely to be of higher energy relative to the experiments described in this study.

## Conclusions

In this study, we have performed a series of attrition experiments on the mineral, olivine. Our experiments have informed on the attrition potential within two different energy environments, which are both relevant to different parts of the kimberlite system (Fig. 1). The tumbling mill resembles relatively low energy environments where the interacting particles are kept in near-continual contact (e.g. sedimentary bed load transport in rivers), whereas the fluidized bed subjects particles to higher energy environments within a gas (e.g. the gas/fluid-rich head of an ascending dyke).

Grain size data and SEM imagery have allowed us to show that abrasion is the dominant form of attrition under our experimental conditions. Grain size reduction predominantly occurs through grain rounding and the simultaneous production of small surface-derived chips. In both environments, the mass of daughter products produced is non-linear with respect to time and can be modelled in the form m_d_/m_0_ = a (1 – e^–bt^) where a and b are the infinite time limit and the attrition rate constant respectively. Due to greater differential particle velocities, these constants are an order of magnitude larger in the fluidized bed relative to the tumbling mill. Finally, we suggest that attrition rate models, like the ones presented here, could be used in the future to “forensically” assess the transport conditions of enigmatic kimberlite processes: e.g. what are the rates and durations of magma ascent?
